# Balance between Fe^IV^–Ni^IV^ synergy and Lattice Oxygen Contribution for Accelerating Water Oxidation

**DOI:** 10.1021/acsnano.4c01718

**Published:** 2024-05-21

**Authors:** Chao Jing, Lili Li, Yi-Ying Chin, Chih-Wen Pao, Wei-Hsiang Huang, Miaomiao Liu, Jing Zhou, Taotao Yuan, Xiangqi Zhou, Yifeng Wang, Chien-Te Chen, Da-Wei Li, Jian-Qiang Wang, Zhiwei Hu, Linjuan Zhang

**Affiliations:** † Key Laboratory of Interfacial Physics and Technology, Shanghai Institute of Applied Physics, Chinese Academy of Sciences, Jialuo Road 2019, Shanghai 201800, P.R. China; ‡ School of Chemistry & Molecular Engineering, East China University of Science and Technology, 130 Meilong Road, Shanghai 200237, P.R. China; § Max Planck Institute for Chemical Physics of Solids, Nöthnitzer Strasse 40, Dresden 01187, Germany; ∥ State Key Laboratory of Crystal Materials and Institute of Crystal Materials, Shandong University, Jinan 250100, P.R. China; ⊥ Department of Physics, National Chung Cheng University, Chiayi 621301, Taiwan, R.O. China.; # National Synchrotron Radiation Research Center, 101 Hsin-Ann Road, Hsinchu 300092, Taiwan, R.O. China; ∇ Zhejiang Institute of Photoelectronics & Zhejiang Institute for Advanced Light Source, Zhejiang Normal University, Jinhua, Zhejiang 321004, P.R. China; ○ University of Chinese Academy of Sciences, Beijing 100049, P.R. China

**Keywords:** water splitting, *in situ* X-ray absorption spectroscopy, *in situ* Raman spectroscopy, intersite hopping, lattice oxygen contribution

## Abstract

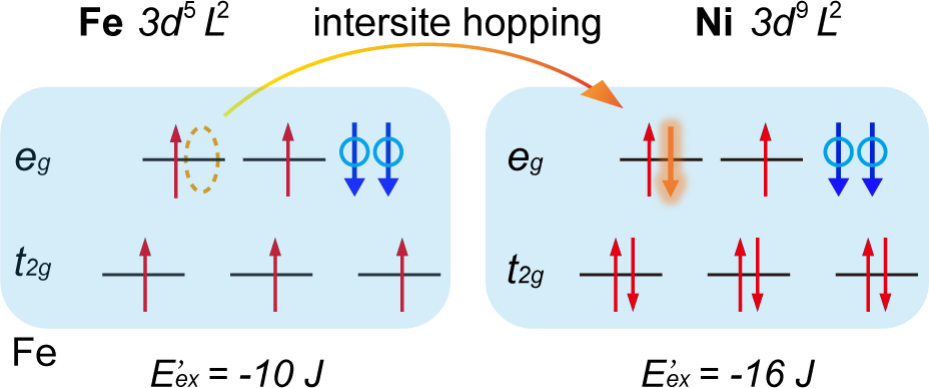

Hydrogen obtained from electrochemical water splitting is the most promising clean energy carrier, which is hindered by the sluggish kinetics of the oxygen evolution reaction (OER). Thus, the development of an efficient OER electrocatalyst using nonprecious 3d transition elements is desirable. Multielement synergistic effect and lattice oxygen oxidation are two well-known mechanisms to enhance the OER activity of catalysts. The latter is generally related to the high valence state of 3d transition elements leading to structural destabilization under the OER condition. We have found that Al doping in nanosheet Ni–Fe hydroxide exhibits 2-fold advantage: (1) a strong enhanced OER activity from 277 mV to 238 mV at 10 mA cm^–2^ as the Ni valence state increases from Ni^3.58+^ to Ni^3.79+^ observed from *in situ* X-ray absorption spectra. (2) Operational stability is strengthened, while weakness is expected since the increased Ni^IV^ content with 3d^8^L^2^ (L denotes O 2p hole) would lead to structural instability. This contradiction is attributed to a reduced lattice oxygen contribution to the OER upon Al doping, as verified through *in situ* Raman spectroscopy, while the enhanced OER activity is interpreted as an enormous gain in exchange energy of Fe^IV^–Ni^IV^, facilitated by their intersite hopping. This study reveals a mechanism of Fe–Ni synergy effect to enhance OER activity and simultaneously to strengthen operational stability by suppressing the contribution of lattice oxygen.

The oxygen evolution reaction (OER) plays a critical role in modern industrial water splitting because of its sluggish kinetics.^1−3^ Therefore, developing efficient, stable, and low cost catalysts for OER are imperative for applications in renewable energy.^4−6^ Recently, highly active and low-cost earth-abundant 3d transition metal (TM) catalysts for industrial applications have garnered growing interest.^7−10^ The 3d TM catalysts with higher metal valence states have been reported to exhibit high OER activity, benefiting from the efficient intermediate adsorption and charge transfer process during the catalytic reaction.^11−13^ For instance, ACoO_3_ (A = Ca and Sr),^14^ CaCu_3_Fe_4_O_12_,^15^ and Li_*x*_NiO_2_ (0 < *x* < 1),^16^ a catalyst with high-valence Ni ions, or created under OER condition, showed excellent OER activity with double ligand holes (3d^*n*^L^2^ with *n* = 6, 7, 8 for Fe^IV^, Co^IV^, and Ni^IV^, respectively, and L^2^ stands for 2 holes at the O 2p orbital) as catalytic active sites. Furthermore, the high metal valence states and formed O 2p holes raise the barrier for electron removal from metal sites, facilitating the oxidation of oxygen sites.^17−19^ The enhanced activity of oxygen sites would promote OER pathways involving lattice oxygen, including the lattice-oxygen-vacancy-site mechanism (LOV) and metal-and-lattice-oxygen-vacancy-site mechanism (MLOV) as shown in Scheme 1.^20−23^ These pathways involving lattice oxygen are considered as more efficient OER routes than the conventional adsorbate evolution mechanism (AEM, Scheme 1).^18,24,25^ However, in pathways involving lattice oxygen oxidation related to high oxidation state, the massive oxygen vacancies can be created under high overpotentials.^26^ This can lead to the destabilization of the catalyst structure, thereby a decrease in OER activity.^14,27^ Therefore, the strategies to enhance the OER performance in 3d TM catalysts for industrial applications involve a balance between valence states of 3d TM and lattice oxygen stability.

**Scheme 1:**
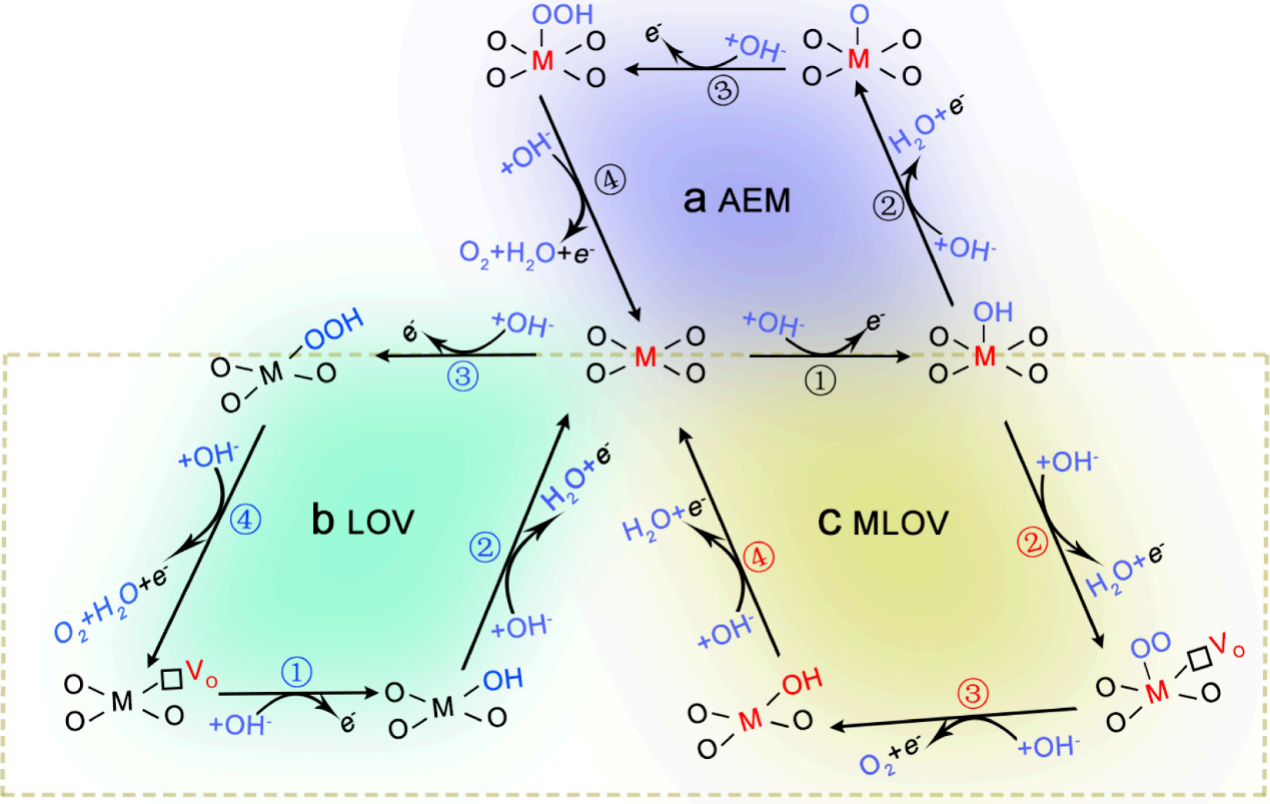
Illustration of the Oxygen Evolution Reaction (OER) Pathways (a) Adsorbate evolution mechanism (AEM). (b) Lattice-oxygen-vacancy-site mechanism (LOV). (c) Metal-and-lattice-oxygen-vacancy-site mechanism (MLOV).

Low-cost layered 3d TM hydroxides/(oxy)hydroxide with high catalytic capability are promising catalysts in industrial water splitting.^28,29^ These low-dimensional nanosheet TM catalysts showed excellent structural flexibility which enables facile structural reconstructions and sufficient surface oxidation to generate abundant active species.^30,31^ A critical role of high-valence state of Ni^IV^ and Fe^IV^ in the high OER activity in Ni–Fe hydroxide is well-known in the literature.^32−37^ On the one side, multimetal catalysts are verified to be more OER efficient than single metal, as generally called synergy effects.^38,39^ On the other hand, lattice oxygen involves in OER activity due to the high oxidation state of Fe^IV^–Ni^IV^ with high content O 2p holes.^40−45^ However, Ni–Fe hydroxide faces challenges in long-term stability under industrial settings since such high valence state ions are very unstable due to too much unstable O 2p.^27,46−48^ Here, we introduced Al, with a stable +3 valence state under high overpotentials, into the Ni–Fe hydroxide to enhance the stability and activity of the catalysts.^49,50^*In situ* X-ray absorption spectra (XAS) suggested a positive correlation between OER activity (381, 277, and 238 mV at 10 mA cm^–2^) and the valence state of Ni (+3.50, + 3.58 and +3.79) in the sequence Ni hydroxide, Ni–Fe hydroxide, and Al doped Ni–Fe hydroxide, while our *in situ* Raman spectroscopy indicated a suppressed lattice oxygen contribution to OER activity. These results demonstrate that Al-doped Ni–Fe hydroxide not only resulted in an increased Ni(IV) content with double ligand holes for enhancing OER activity, but also suppressed the lattice oxygen oxidation, contributing to improved operational stability.

## Results and Discussions

### Theoretical Prediction

**Figure 1:**
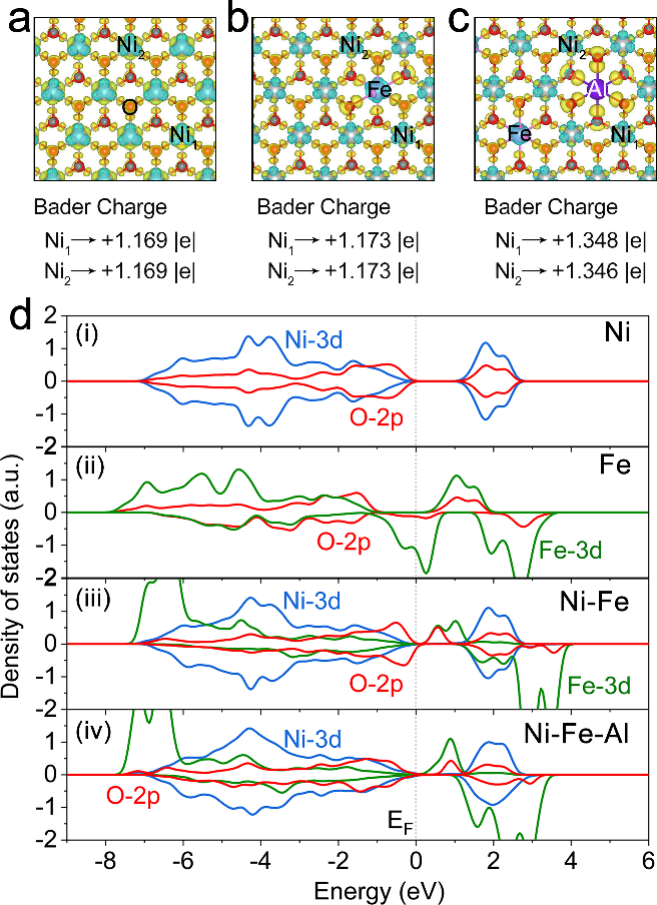
Calculation of Bader charge and projected density of states (PDOS). (a–c) Electron localization function of the Ni, Ni–Fe, and Ni–Fe–Al oxides. (d) Plots of calculated PDOS of the Ni, Fe, Ni–Fe, and Ni–Fe–Al oxides.

To understand the influence of Al doping on the activity of metal and lattice oxygen sites of Ni–Fe hydroxide, we calculated the electronic structure of Ni, Ni–Fe, and Ni–Fe–Al nanosheet hydroxides based on density functional theory (DFT) simulations (calculation details are provided in the Supporting Information (SI)). Owing to the existence of Ni^IV^ and Fe^IV^ after the OER reported in previous reports, layered Ni^IV^ and Fe^IV^ oxides were used as the DFT calculation models.^16,35,37^Figure 1a–c illustrates the Bader charge of Ni ions in the three catalysts. According to the DFT calculations, the charge of Ni ions increased only a little from 1.169 to 1.173 by involvement of Fe but sharply increased to 1.349 by further involvement of Al, since the Al ion has a much higher fourth ionization energy (electron removal): E4 (Al) = 120 eV, than Ni ion: E4 (Ni) = 54.9 eV and Fe ion: E4 (Fe) = 54.8 eV.

Subsequently, we analyzed the projected electronic density of states (PDOS) of Ni, Fe, Ni–Fe, and Ni–Fe–Al oxides, as shown in Figure 1d. As evidenced in Figure 1d-i, Ni^IV^ oxide exhibits an obvious band gap at the Femi level (E_F_), indicating an insulating property. Conversely, Fe^IV^ oxide demonstrates metallic conductivity without band gaps at the Fermi level, as depicted in Figure 1d-ii. The density state at Fermi energy has a considerable O 2p character because of negative charge transfer, which is facile to the lattice oxygen involved pathway. However, the active lattice oxygen atoms possessing numerous O 2p holes are not stable under vigorous OER conditions, resulting in the dissolution of catalysts.

In the case of Ni–Fe oxide (Figure 1d-iii), the narrow band gap occurs near the *E*_F_ and then the transport property falls between those of NiO_2_ and FeO_2_. This narrow band gap has the potential to facilitate electron transfer during the OER process, highlighting Ni–Fe synergistic effect.^51^ Upon further Al doping, a band gap becomes slightly broader than that in Ni–Fe hydroxide. We hypothesize that this modest band gap not only maintains the Ni–Fe synergistic effect but also modulates the stability of lattice oxygen, potentially suppressing the dissolution of the catalysts under high potentials. Hence, an appropriate band gap of the catalyst critically influences OER activity, serving as a descriptor for OER catalysts.^27^

Now, we further discuss the relation between metal–oxygen covalence and OER activity.^18^ In the case of Fe substitution (Figure 1d-iii), an unoccupied antibonding O orbital emerges at *E*_F_, which may promote the adsorption of intermediates. In addition, at ca. 1 eV above *E*_F_, the interaction between Fe 3d and O 2p is relatively weak. However, in the presence of Al, the Fe-3d-O-2p and Ni-3d-O-2p covalent mixings are markedly strengthened, but the density state, especially O 2p at *E*_F_, weakens (Figure 1d-iv), reflecting reduced unstable O 2p holes,^48^ in other words, enhanced operational stability. Thus, we predict that doping with Al could further increase both the Fe–O and Ni–O covalence. This suggests an enhanced adsorption capability of oxygen-containing intermediates on the metal sites. Therefore, Al doped Ni–Fe oxide increased the Ni(Fe)–O covalence and contributed to the development of an appropriate band gap, which is essential for regulating the metal valence state and lattice oxygen activity.

### Structural Characterization of the As-Prepared Catalysts

**Figure 2:**
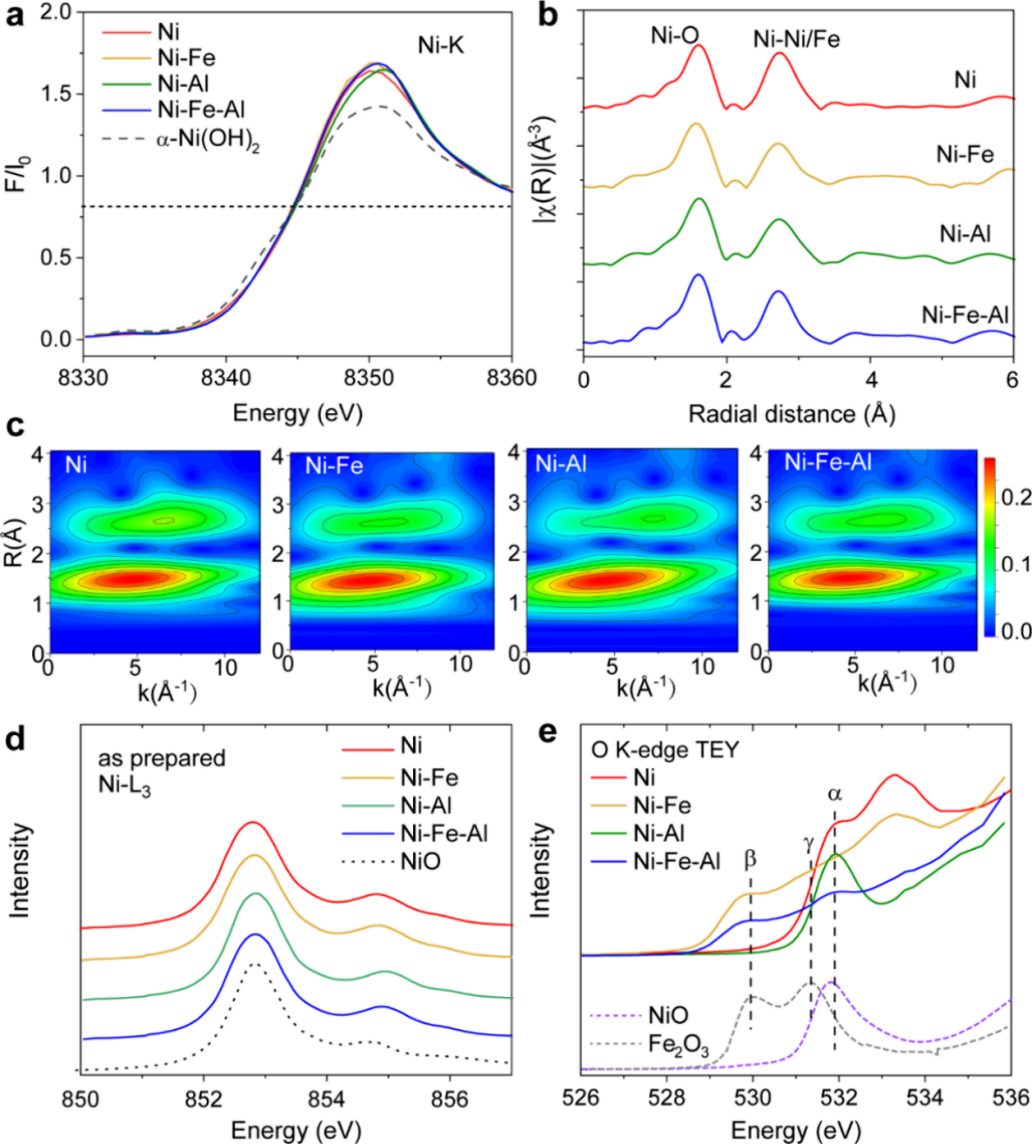
X-ray absorption spectroscopy (XAS) characterization of the as-prepared catalysts. (a) Ni K-edge X-ray absorption near edge structure (XANES) spectra of the pristine Ni, Ni–Fe, Ni–Al, and Ni–Fe–Al hydroxide catalysts with a reference sample of commercial Ni(OH)_2_. (b) Fourier transformed K^2^χ(R) Ni K-edge extended X-ray absorption fine structure (EXAFS) of the four catalysts. (c) Wavelet transforms of the EXAFS signal (distance R (Å) (not corrected for phase shift) vs photoelectron wavenumber k (Å^–1^) of Ni, Ni–Fe, Ni–Al, and Ni–Fe–Al hydroxides. (d) Ni L_3_-edge XAS of pristine Ni, Ni–Fe, Ni–Al, and Ni–Fe–Al hydroxide catalysts with a reference sample of commercial NiO. (e) O K-edge XAS of pristine Ni, Ni–Fe, Ni–Al, and Ni–Fe–Al hydroxide catalysts with reference samples of commercial NiO and Fe_2_O_3_.

Ni, Ni–Fe, Ni–Al, and Ni–Fe–Al nanosheet hydroxides were fabricated using an electrodeposition method described in previous reports.^47,52^ The doping ratios of Fe and Al were optimized so that Ni_75_Fe_25_ hydroxide and Ni_75_Fe_25_Al_10_ hydroxide showed the best OER performance (Figure S1a,b in the SI). All the definitions of the samples are according to the precursor solutions. As the doping of Ni hydroxide with Al results in a decrease in the OER activity, Ni_90_Al_10_ hydroxide was prepared for further analysis. The corresponding elemental ratios of the catalysts, as detected by inductively coupled plasma mass spectrometry (ICP-MS), are listed in Table S1 of the SI. Transmission electron microscopy (TEM) and scanning electron microscopy (SEM) characterizations are depicted in Figures S2 and S3, revealing low-crystallinity structures in all four compounds. Energy-dispersive spectroscopic analysis (Figure S2) indicated the homogeneous distribution of the analyzed elements. It is well-known that the electronic structures and local coordination of Ni in the pristine sample can be explored by X-ray absorption near edge structure (XANES)^53^ and the extended X-ray absorption fine structure (EXAFS),^15^ respectively. In the Ni K-edge region (Figure 2a), the valence states of Ni are close to +2 in all of the electrodeposited hydroxides, as compared with those of commercial Ni(OH)_2_. In the EXAFS region (Figure 2b,c), all four catalysts exhibited two main peaks corresponding to the Ni–O and Ni–Ni/Fe scattering paths. EXAFS simulations at the Ni K-edge could provide bonding length (*R*) and coordination numbers (*N*) of coordination spheres around Ni. A first coordination shell fitting was employed to obtain the structural parameters of the four samples (Figure S4 and Table S2). All four catalysts presented a saturated O shell (*N* ≈ 6) with an *R*(Ni–O) close to 2.05, consistent with a previous report.^37^Figure S5a exhibits the Fe K-edge XANES of Ni–Fe and Ni–Fe–Al hydroxides with Fe_2_O_3_ as a reference, revealing the Fe^3.2+^ ion in both catalysts.

The valence state^43,54^ and local environment^55^ significantly influence the multiplet spectral features and the energy position observed in the L_3_-edge XAS spectra of transition elements. Ni L_3_-edge XAS (Figure 2d) revealed that the energy positions of all four catalysts were identical to those of NiO, suggesting a Ni^2+^ state derived from NiO_6_ octahedral sites.^53^ In addition, pre-edge peaks of O K-edge below 533 eV are from unoccupied O 2p orbitals hybridized with Ni/Fe 3d states and energy position shifts to lower energy with an increase in the valence state of 3d transition elements.^56−59^ The O K-edge spectra in Figure 2e for the four catalysts exhibit peaks at approximately 531.9 eV (peak α), similar to those of NiO with a Ni^2+^ state, in agreement with the Ni K- and L_3_-edge XAS results. Upon substitution with Fe, the Ni–Fe and Ni–Fe–Al hydroxides yielded two peaks at 529.7 (peak β) and 531.3 eV (peak γ), indicating an Fe valence exceeding +3 compared with Fe_2_O_3_. Figure S5b presents the Fe L_3_-edge XAS, thus supporting the presence of the Fe^(3+δ)+^ state in the Ni–Fe and Ni–Fe–Al hydroxides, in agreement with the O K-edge XAS results in Figure 2e.

### Catalyst OER Performance Analysis

**Figure 3:**
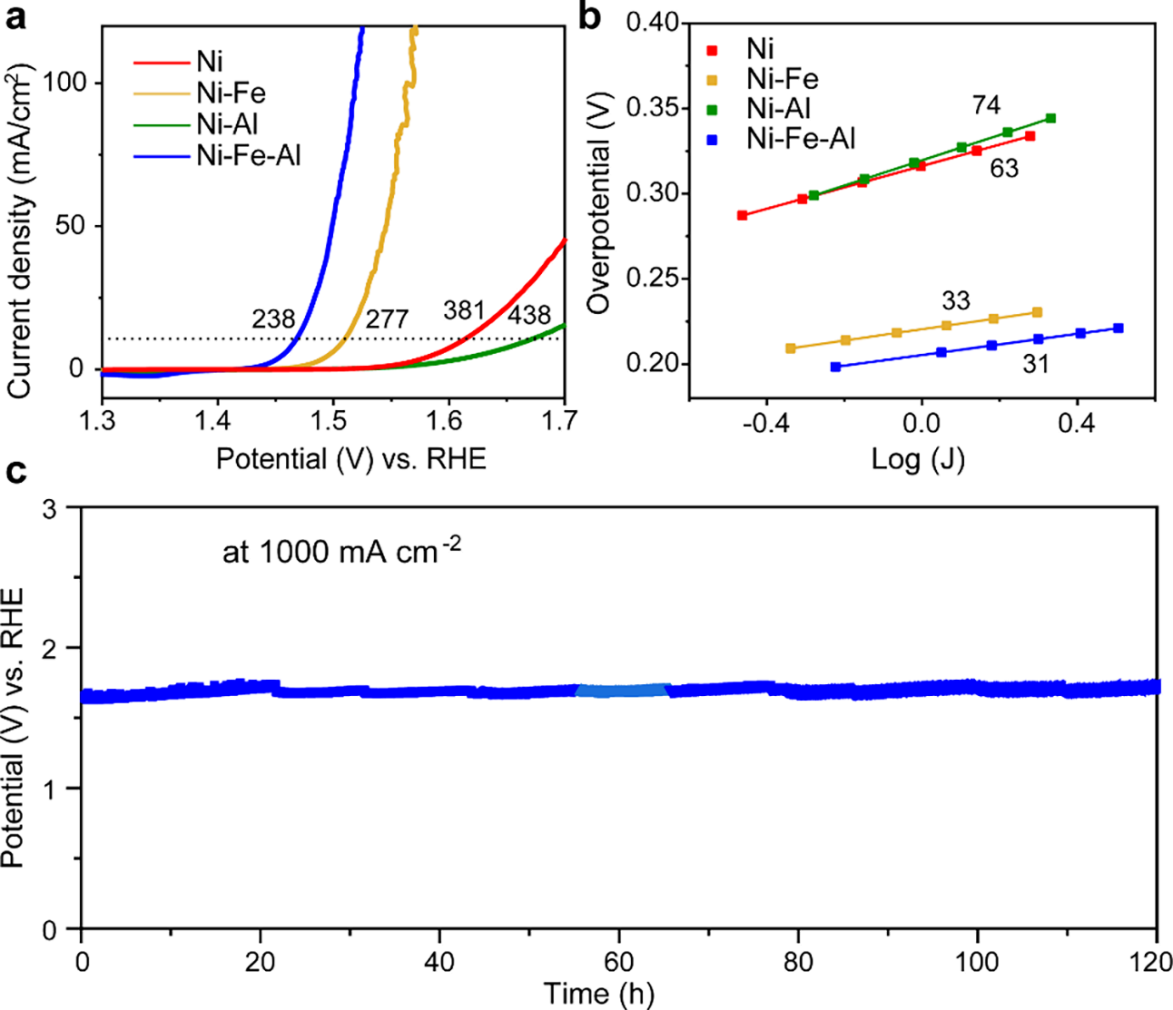
Electrochemical performance of the Ni, Ni–Fe, Ni–Al, and Ni–Fe–Al hydroxide catalysts in 1 M KOH electrolyte solution. (a) Linear sweep voltammetry curves (scan rate: 5 mV s^–1^, *iR* compensation: 90%), (b) Tafel slopes measured at steady states (*iR* compensation: 100%), and (c) stability test at 1000 mA cm^–2^ using Ni foam as the working electrode (*iR* compensation: 90%).

The catalysts were electrodeposited on carbon paper working electrodes, and their OER performances were characterized using linear sweep voltammetry (LSV) in 1 M KOH electrolyte solution. The OER overpotentials for Ni, Ni–Fe, Ni–Al, and Ni–Fe–Al hydroxides at a current density of 10 mA cm^–2^ were 381, 277, 438, and 238 mV, respectively (Figure 3a). Compared with Ni hydroxide, Ni–Al hydroxide exhibited a decrease in catalytic capability. Conversely, doping with Fe distinctly enhanced OER catalytic activity owing to the strong Ni–Fe synergistic effect.^60^ Codoping with Fe and Al further improved the OER performance, thus indicating that the inactive Al enhanced the OER process in the presence of Fe. The Tafel slopes in Figure 3b verified the superior properties of Ni–Fe–Al hydroxide, with a value of 31 mV dec^–1^. As shown in Figure S6, Ni–Fe–Al hydroxide exhibited the highest electrochemical active surface area (ECSA) with a double-layer capacitance (*C*_dl_) of 21.4 mF cm^–2^. Notably, Ni–Fe hydroxide and bare Ni foam showed poor stability during the long-term OER process (Figures S7–S8), whereas Ni–Fe–Al hydroxide demonstrated excellent performance even under extremely high currents (Figure 3c). A decrease of only 3% was observed after 120 h of operation at a current density of 1000 mA cm^–2^, highlighting its potential for sustainable industrial hydrogen production. In addition, the concentration of dissolved Al ions of Ni–Fe–Al hydroxide during the OER process was determined by ICP-MS as shown in Figure S9. This analysis underscores that the dissolution of Al ions is primarily associated with the structural reconstruction process within the first hour. Following a 5 h OER process, the catalyst gradually achieves stabilization.

### Catalysts’ Structural Reconstruction Process

**Figure 4:**
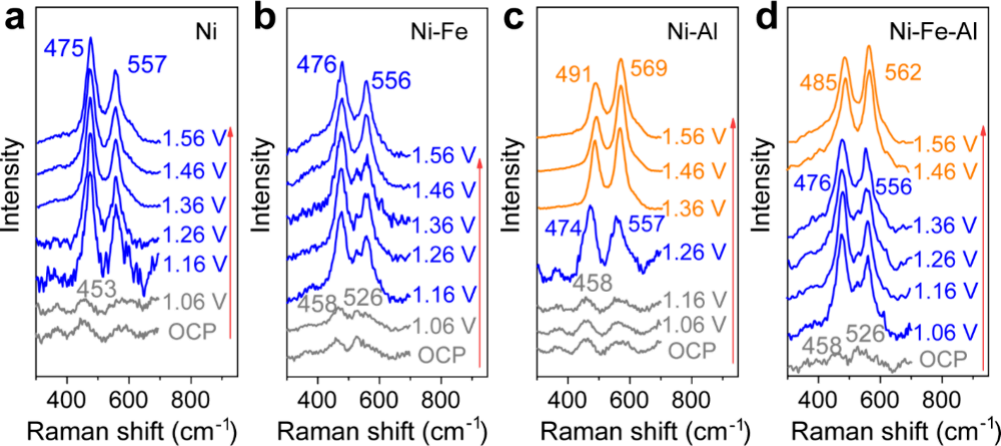
Raman spectroscopy. (a–d) *In situ* Raman spectra of the Ni (a), Ni–Fe (b), Ni–Al (c), and Ni–Fe–Al (d) hydroxide catalysts at various potentials (vs RHE) in 0.1 M KOH electrolyte.

*In situ* Raman spectroscopy was conducted to investigate the dynamic OER process (Figure 4).^11,61,62^ Herein, 0.1 M KOH was used as the electrolyte to prevent signal attenuation from the vigorous bubbling that may occur in the 1 M KOH solution under high-OER-potential conditions. Previous reports investigated the *in situ* Raman spectroscopy of Ni(OH)_2_ using various KOH concentrations and yielded similar results.^31^ The *in situ* Raman spectra of Ni hydroxide in Figure 4a exhibit a small peak at 453 cm^–1^ at the open-circuit potential, which is attributed to Ni(II)-O vibrations.^63^ At 1.16 V, two bands appear at 475 and 557 cm^–1^ with an intensity ratio of 1:0.7, which are attributed to γ-NiOOH.^63,64^ At increasing potentials, the Raman peaks remained unchanged, indicating γ-NiOOH as the active species. Ni–Fe hydroxide demonstrated a similar reaction pathway, as shown in Figure 4b. The as-prepared Ni–Fe hydroxide displayed two peaks at 458 and 526 cm^–1^, corresponding to the Ni(II)-O and Fe(III)-O vibrations.^65^ When the potential reached 1.36 V, the characteristic peaks of γ-NiOOH at 476 and 556 cm^–1^ emerged. Both the Raman peak position and intensity ratio of Fe-doped γ-NiOOH showed slight alternations in comparison to pure γ-NiOOH, which could be ascribed to the lattice distortion generated by Fe substitution.^63^ Thus, γ-NiOOH is considered the active phase in Ni–Fe hydroxide, and the significant enhancement in the OER activity was attributed to the Ni–Fe synergic effect.

The Raman spectra of Ni–Al hydroxide display notable differences compared with those of Ni and Ni–Fe hydroxides, as depicted in Figure 4c. At 1.26 V, two peaks belonging to γ-NiOOH appear at 474 and 557 cm^–1^. However, upon reaching 1.36 V, the Raman peaks shifted to 491 and 569 cm^–1^ with an intensity ratio of 0.8:1. It is postulated that the leaching of Al atoms in the lattice results in a structural reconstruction to a new phase. This phase was assigned to β-type NiOOH according to previous research.^46^ Interestingly, after switching off the applied potential, the Raman peaks of NiOOH shifted from 491 to 481 cm^–1^ and from 569 to 562 cm^–1^ within 2 h, as illustrated in Figure S10a. As earlier studies suggested that the valence of the real Ni-sites in the OER exceeds +3, the NiOOH bands at 491 and 569 cm^–1^ are assumed to be overcharged β-NiOOH with partial Ni(IV) content. When the applied potential is switched off, Ni(IV) proved difficult to retain, thus leading to a reduction to β-Ni(III)OOH with Raman bands at 481 and 562 cm^–1^.

In the case of the Ni–Fe–Al hydroxide, the *in situ* Raman spectra exhibited features of both the Ni–Fe and Ni–Al hydroxides, as depicted in Figure 4d. The spectra of the as-prepared Ni–Fe–Al hydroxide exhibited two weak bands at 458 and 526 cm^–1^, attributed to the Ni(II)-O and Fe(III)-O vibrations. At 1.06 V, the typical bands of γ-NiOOH appeared at 476 and 556 cm^–1^. At 1.46 V and higher voltages, two peaks emerge at 485 and 562 cm^–1^, suggesting a partial transformation of the catalyst from γ-NiOOH to β-NiOOH. We assume that the presence of Fe hindered the dissolution of Al in comparison to Ni–Al hydroxide, thus resulting in the suppressed formation of β-NiOOH at higher potentials. The element ratio of the catalysts after the OER (Table S1) further confirms that Fe substitution restricts the dissolution of Al. We hypothesize that a mixture of γ-NiOOH and β-NiOOH formed upon oxidation of the Ni–Fe–Al hydroxide. When the applied potential was switched off, the bands in the Raman spectra shifted to 477 and 558 cm^–1^, as shown in Figure S10b. This shift signifies the transformation of Ni(IV)OOH_*x*_ to Ni(III)OOH.

**Figure 5:**
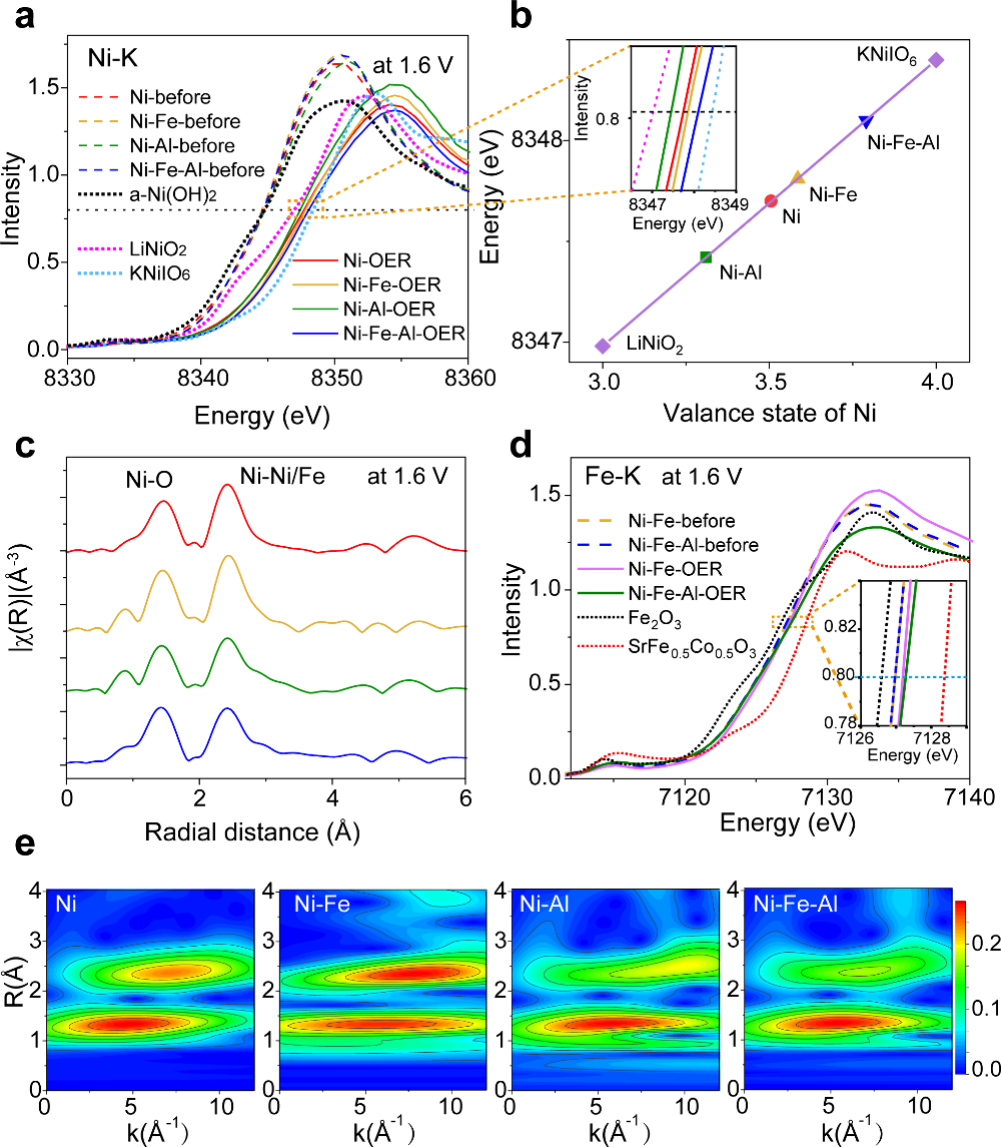
*In situ* XAS characterization of the catalysts during the OER process in 0.1 M KOH electrolyte. (a) *In situ* Ni K-edge XANES spectra of the Ni, Ni–Fe, Ni–Al, and Ni–Fe–Al hydroxide catalysts at the open-circuit potential and 1.6 V with reference samples of LiNi(III)O_2_ and KNi(IV)IO_6_. (b) Calculated valence states of Ni in Ni, Ni–Fe, Ni–Al, and Ni–Fe–Al hydroxides at 1.6 V (the inset shows the zoomed-in spectra of (a)). (c) *In situ* Fourier transformed K^2^χ(R) Ni K-edge EXAFS of the four catalysts at 1.6 V, and (d) *in situ* Fe K-edge XAS spectra of Ni–Fe and Ni–Fe–Al hydroxides with Fe(III)_2_O_3_ and SrFe(IV)_0.5_Co_0.5_O_3_ references. (e) Wavelet transforms of the EXAFS results (distance R (Å) (not corrected for phase shift) vs photoelectron wavenumber k (Å^–1^) of Ni, Ni–Fe, Ni–Al, and Ni–Fe–Al hydroxides at 1.6 V. The applied potentials are referenced to the RHE scale.

To reveal the changes in the electronic structure of the catalysts during the OER process, *in situ* Ni K-edge XAS was performed in 0.1 M KOH as depicted in Figure 5. At 1.6 V, as illustrated in Figure 5a,b, the valence states of Ni within the four catalysts were estimated to be +3.50 (Ni), +3.58 (Ni–Fe), +3.31 (Ni–Al), and +3.79 (Ni–Fe–Al) with references of LiNi(III)O_2_ and KNi(IV)IO_6_. A positive correlation between the OER performance and the valence state of Ni is evident. Figure 5c,e depicts the *in situ* Ni K-edge EXAFS at 1.6 V, and the simulated results are displayed in Figure S11 and Table S3. The two peaks in Figure 5c originate from the Ni–O and Ni–Ni/Fe shells. Notably, Ni–Fe–Al hydroxide exhibited the shortest Ni–O bonding length (approximately 1.87 Å), indicative of the highest valence state of Ni. Figure 5d presents the *in situ* Fe K-edge XAS of Ni–Fe and Ni–Fe–Al hydroxides at 1.6 V, with Fe(III)_2_O_3_ and SrFe(IV)_0.5_Co_0.5_O_3_ as references. The spectra indicate an increased Fe valence state in both the Ni–Fe and Ni–Fe–Al hydroxides during the OER, thus suggesting the existence of Fe^IV^. Notably, the valence state of Fe exhibited a more significant increase in Ni–Fe^3.38+^–Al hydroxide compared with the Ni–Fe^3.32+^ hydroxide. Thus, based on the XAS results, we hypothesized that the doping of Ni–Fe hydroxide with Al(III) ions could further elevate the valence states of both Ni and Fe, thereby improving the OER performance *via* an enhanced metal synergistic effect. On the contrary, to decrease the Ni(IV) content, Zr^4+^ ion-doped Ni–Fe hydroxide was prepared, which exhibited decreased OER activity with an overpotential of 290 mV at 10 mA cm^–2^ as shown in Figure S12 in the SI.

*Ex situ* Ni L_3_-edge, O K-edge, and Fe L_3_-edge XAS were employed to characterize the electronic structure of the four catalysts after the OER process, as illustrated in Figure S13. Ni(III/IV)OOH_*x*_ proved to be unstable in air and was readily reduced to Ni(II). As a result, the *ex situ* Ni L_3_-edge and O K-edge XAS spectra of the four catalysts revealed the presence of a mixture of Ni(III) and Ni(II) compared with NiO and Ni(III)OOH references, as shown in Figure S13a–c. Furthermore, according to the Fe L_3_-edge spectra in Figure S13d, the Ni–Fe–Al hydroxide exhibited an approximate 0.15 eV energy shift after the OER, thus suggesting that the Al substitution may promote the Fe–O covalence. In addition, high-resolution TEM (Figure S14) and SEM (Figure S15) characterizations of the four catalysts post-OER indicate structural stability and homogeneous elemental distribution.

### Lattice Oxygen Exchange Process

**Figure 6:**
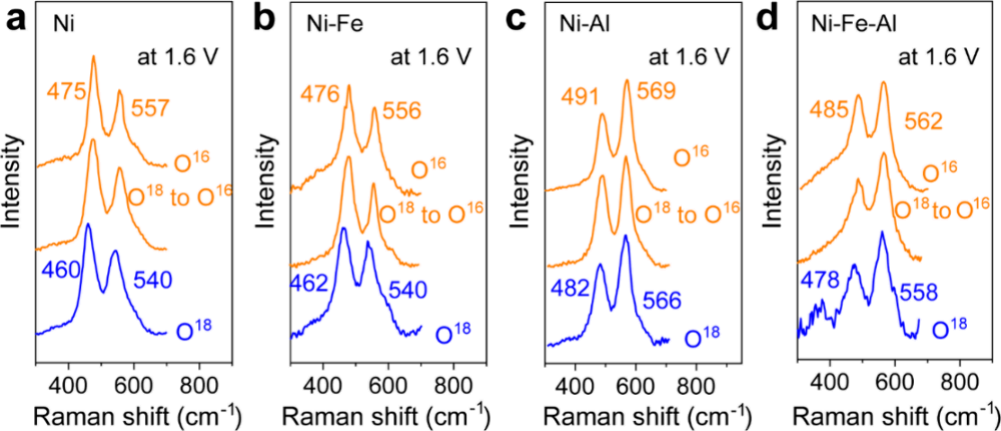
Raman spectroscopy of the O^18^-labeled catalysts. *In situ* Raman spectra of the Ni (a), Ni–Fe (b), Ni–Al (c), and Ni–Fe–Al (d) hydroxide catalysts at 1.6 V vs RHE in 0.1 M KO^16^H and KO^18^H.

*In situ* Raman spectroscopy of the prepared catalysts was performed in a KO^18^H solution to elucidate the OER mechanism. As depicted in Figure 6a, the γ-NiOOH labeled with O^18^, formed from Ni hydroxide, exhibited Raman peaks at 460 and 540 cm^–1^, differing by 15 and 17 cm^–1^ from those in KO^16^H solution. Subsequently, *in situ* Raman spectroscopy of the O^18^ labeled γ-NiOOH was conducted in KO^16^H solution at 1.6 V. The Raman peaks in the spectra shifted back to 475 and 557 cm^–1^; this is the same outcome as that obtained when the KO^16^H solution was used, thus indicating the presence of the lattice oxygen involved mechanism in Ni hydroxide as reported previously.^31,45,66^ The O^18^ labeled Ni–Fe oxyhydroxide exhibited two Raman bands at 462 and 540 cm^–1^ (Figure 6b). Like those of Ni hydroxide, these two Raman peaks shifted to 476 and 556 cm^–1^ post-OER in KO^16^H solution, suggesting that they occurred through a lattice oxygen pathway. However, in the case of Ni–Al and Ni–Fe–Al hydroxides in KO^18^H solution, the shift of the Raman peaks corresponding to the formed O^18^-labeled NiOOH, compared with those obtained in KO^16^H solution, was less than 10 wavenumbers, as shown in Figure 6c,d. Therefore, we assumed that the substitution with Al can result in the inhibition of the oxygen exchange between the lattice oxygen and electrolyte oxygen compared with the exchange observed in Ni and Ni–Fe hydroxides. Upon switching to the KO^16^H electrolyte, the peaks in the spectra of the Ni–Al and Ni–Fe–Al oxyhydroxides partially labeled with O^18^ shifted to the peak positions of O^16^–NiOOH. Consequently, we assumed a suppression of the oxygen activity with decreased lattice oxygen contribution in the Ni–Al and Ni–Fe–Al hydroxides.

### OER Mechanism

**Figure 7:**
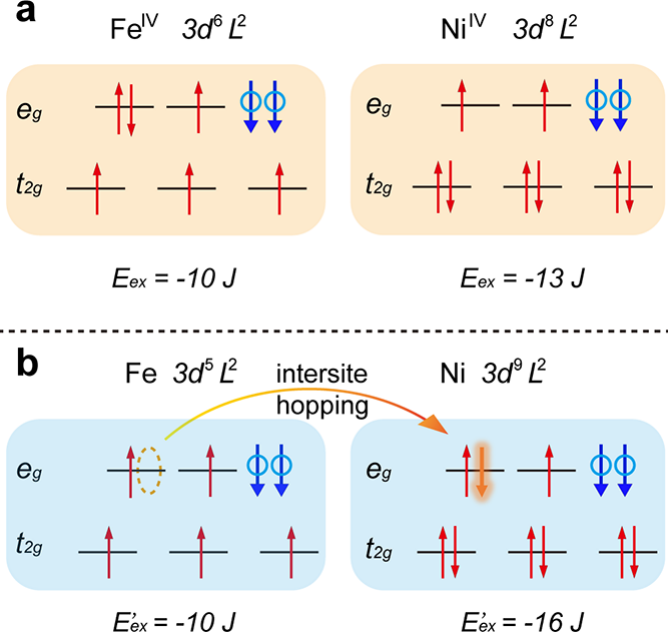
Energy diagrams: Fe^IV^ (3d^6^L^2^)/Ni^IV^ (3d^8^L^2^) pair in the initial state (a) and after intersite hopping (b).

The OER overpotentials at 10 mA cm^–2^ of the prepared hydroxide catalysts exhibited the following trend: Ni (381 mV) ≪ Ni–Fe (277 mV) < Ni–Fe–Al (238 mV). This order corresponds to an increase in the valence states of Ni from Ni^2+^ in the pristine catalysts to Ni^3.50+^, Ni^3.58+^, and Ni^3.79+^. We assumed that Ni sites function as the main OER active species in all prepared catalysts. Al-doped Ni–Fe hydroxide enhances the valence states of Ni, promoting a more pronounced Ni–O covalence. We examined the exchange interactions between the 3d^6^L^2^ of the Fe^IV^ ion^41^ and 3d^8^L^2^ of the Ni^IV^ pair^16^ in Figure 7 and detected ferromagnetic exchange interactions. Before hopping, the total energy was −23 J_ex_ (neglecting a small difference in crystal field energy), whereas after hopping, the total energy was −26 J_ex_. Intersite hopping led to a significant increase in the exchange energy (3 J_ex_ or approximately 3 eV). Therefore, the Fe^IV^/Ni^IV^ pair exhibited higher transport properties than any other pair. Al doping leads to high Ni^IV^ content under the OER conditions, thereby increasing the possibility of intersite hopping, namely, the transport properties. This clearly explains both the synergistic effect between metal ions with high-oxidation states and the lattice oxygen oxidation for boosting the OER.

**Figure 8:**
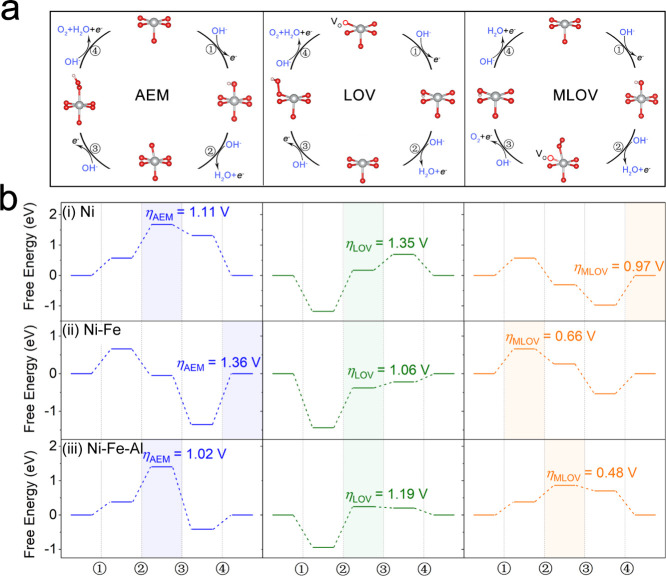
OER mechanism. (a) Schematic showing the OER mechanisms of AEM, LOV, and MLOV. (b) Free energies for the OER steps on (i) NiOOH_0.5_, (ii) Fe-doped NiOOH_0.5_, and (iii) Fe–Al-doped NiOOH_0.5_ in the AEM, LOV, and MLOV pathways, respectively.

DFT calculations were subsequently performed to model the Gibbs free-energy difference (Δ*G*) for the reaction steps in the AEM, LOV, and MLOV pathways of Ni, Ni–Fe, and Ni–Fe–Al hydroxides shown in Figure 8. Herein, Ni oxyhydroxides with Ni^3.50+^ ions were employed as the bulk structure, and Ni ions functioned as the active sites based on our XAS results. In Figure 8, the three catalysts exhibited the lowest OER overpotentials in the MLOV route. Notably, both the LOV and MLOV mechanisms involve lattice oxygen contributions; however, the calculated overpotential for MLOV is significantly lower than that for LOV. This result indicates an adsorption preference of OER intermediates on metal rather than O sites.^16,21^ In the case of NiOOH_0.5_ (Figure 8b-i), the MLOV route exhibited an overpotential (η) of 0.97 V. Additionally, the formation of M-O* represents the rate-determining step (RDS) (step 4), thus, indicating inefficient charge transfer at the oxygen site. The high-OER overpotentials calculated for NiOOH_0.5_ are consistent with the low-OER activity observed experimentally.

In the case of Fe-doped NiOOH_0.5_ (depicted in Figure 8b-ii), the calculated overpotential in the MLOV pathway was 0.66 V, with the RDS shifting to the adsorption of OH^–^ on the metal sites (step 1). In the PDOS simulation in Figure 1d, the band gap near the Femi level of the Ni–Fe oxide is dramatically reduced, indicating the occurrence of O 2p holes, which facilitates the electron transfer on the oxygen sites. We inferred that the activated lattice oxygen decreases the Δ*G* for the dehydrogenation of M–OH to form M-O*. Consequently, the RDS shifts from step 4 to 1, thus contributing to a reduction in the OER overpotential.

Figure 8b-iii illustrates that the MLOV process of Fe–Al codoped NiOOH_0.5_ exhibited a significantly lower overpotential than Fe-doped NiOOH_0.5_ and NiOOH_0.5_. The Δ*G* of step 1, corresponding to the OH^–^ adsorption on the Ni sites, decreased from 0.66 V for Fe-doped NiOOH_0.5_ to 0.38 V. This reduction in Δ*G* could be attributed to the enhanced Ni–O covalence facilitated by the doping of Al ion (Figure 1). The formation of Ni–V_O_ is the RDS in the MLOV route (step 2), in good agreement with Raman spectral results, indicating that Al doping suppressed lattice oxygen activity. Thus, compared with Fe-doped NiOOH_0.5_, Fe–Al codoping enhanced metal oxygen covalence and suppressed lattice oxygen activity, leading to a shift in the RDS in the MLOV pathway (from the stage of adsorption of OH^–^ intermediates to the formation of oxygen vacancies). This balance between the synergistic effect of metal sites and the lattice oxygen site stability demonstrates an efficient strategy for the future fabrication of TM OER catalysts.

## Conclusions

In summary, we have investigated the impact of Al doping in Ni–Fe hydroxide on OER catalytic activity. The OER overpotentials for Ni, Ni–Fe, and Al-doped Ni–Fe hydroxide at the current density of 10 mA cm^–2^ strongly decreases from 381 mV, to 277 mV, and further to 238 mV. The *in situ* XANES spectra revealed that under OER condition the contents of Ni^IV^ ions with double ligand holes 3d^8^L^2^ increases from 50% to 58%+ and further to 79%, while Fe ion has a valence state of +3.3 in both Ni–Fe, and Al-doped Ni–Fe hydroxide. The relation between the OER activity and the contents of Ni^IV^ can be interpreted as a significant gain in exchange energy upon Fe^IV^–Ni^IV^ intersite hoping. Moreover, *in situ* Raman spectroscopy demonstrated that Al doping on Ni–Fe hydroxide suppressed the lattice oxygen contribution, which is responsible for the enhanced operational OER stability. These results interpret both the enhanced OER activity by an increase in the content of Ni^IV^ and enhanced stability by a suppression of lattice oxygen contribution. Our study promotes the understanding of the OER mechanisms through the simultaneous modulation of the metal- and lattice oxygen-site activity, which is beneficial for the development of advanced Ni-based OER catalysts for industrial water splitting applications.

## Materials and Methods

### Sample Preparation

All reagents were of analytical grade, including Ni(NO_3_)_2_·6H_2_O, Fe_2_(SO_4_)_3_, Al(NO_3_)_3_·9H_2_O, Zr(NO_3_)_4_, KNO_3_, KOH, and H_2_O^18^(99%), which were purchased from Adamas-beta. Ultrapure water (18.2 MΩ·cm) was produced using the Milli-Q apparatus from Millipore (USA). The carbon paper was from Toray (Japan). The Ni foam and glass carbon electrodes were from Sinero Technology (Suzhou, China). The graphite, Pt and Hg/HgO electrodes were from CH Instruments (USA). Nickel hydroxide and its derivatives were synthesized *via* electrochemical deposition on both a glass carbon electrode (for Raman spectroscopy) and carbon paper electrode. The catalysts were deposited by applying a potential of −0.9 V vs Ag/AgCl for 150s in the following electrolytes: 0.3 M KNO_3_ and 0.03 M Ni(NO3)_2_ for Ni hydroxide; 0.3 M KNO_3_, 0.0225 M Ni(NO_3_)_2_ and 0.00375 M Fe_2_(SO_4_)_3_ for Fe-doped Ni hydroxide; 0.3 M KNO_3_, 0.027 M Ni(NO_3_)_2_ and 0.003 M Al(NO_3_)_3_ for Al-doped Ni hydroxide; 0.3 M KNO_3_, 0.0225 M Ni(NO_3_)_2_, 0.00375 M Fe_2_(SO_4_)_3_ and 0.003 M Al(NO_3_)_3_ for Fe–Al codoped Ni hydroxide, 0.3 M KNO_3_, 0.0225 M Ni(NO_3_)_2_, 0.00375 M Fe_2_(SO_4_)_3_ and 0.003 M Zr(NO_3_)_4_ for Fe–Zr codoped Ni hydroxide.

### Electrochemical Measurements

Three-electrode electrochemical measurements were conducted on a Metrohm Autolab workstation (PGSTAT 302N system) and a CHI 660D electrochemistry station (for *in situ* Raman spectroscopy, CH Instruments). The electrochemical tests were conducted at room temperature in a Teflon cell with an Fe-free 1 M KOH solution saturated with oxygen. Prior to each measurement, the electrochemical cell was cleaned with H_2_SO_4_. The carbon paper and Ni foam were 1 × 1 cm with catalysts deposited on a single side. A graphite electrode and a Hg/HgO electrode (electrolyte: 1 M KOH) served as the counter and reference electrode, respectively. The electrochemical behavior was evaluated after multiple cyclic voltammetry scans, a minimum of 20 cycles, were conducted, until a steady state was reached. Potentials were converted to the reversible hydrogen electrode (RHE) scale using the following formula: *E*_RHE_ = *E*_Hg/HgO_ + 0.059 × pH + 0.097 V, with all reported potentials referenced to the RHE scale unless specified differently. To correct the voltage loss resulting from factors including the low electrolytic conductivity of the electrolyte and the distance between the reference and working electrode, *iR* compensation was executed.^67,68^ The uncompensated resistance (*R*_u_) was determined using electrochemical impedance spectroscopy (EIS) conducted at the open circuit potential (OCP). The sinusoidal wave amplitude was measured over a frequency range from 100 kHz to 100 mHz. In the 1 M KOH electrolyte, the resistance *R*_u_ is ca. 2.5 Ω for the catalysts on the carbon paper and ca. 1.5 Ω for the catalysts on the Ni foam. To avoid over *iR* compensation at high-current region, we used an empirical ratio of 90% for LSV and stability test. For the Tafel slope determined at low-current region, 100% *iR* compensation was applied. The ECSA of the catalysts were calculated according to the equation: ECSA = *C*_dl_/*C*_s_, where the constant *C*_s_ is the specific capacitance of the material per unit area under identical electrolyte conditions. The *C*_dl_ values of the catalysts were calculated through the EIS results by equivalent circuit fitting.

### Soft X-ray Absorption Spectroscopy (SXAS)

For SXAS experiments, the catalysts were electrodeposited on carbon paper. The SXAS experiments at the O K-, Ni L_3_-, and Fe L_3_-edges were measured at the National Synchrotron Radiation Research Center in Taiwan (NSRRC, Hsinchu, 11A beamline). O K-edge XAS were measured by total electron yield (TEY) and total fluorescence yield (TFY) modes.^69^

### Hard X-ray Absorption spectroscopy

*Ex situ* Ni K- and Fe K-edge XAS (transmission mode) were measured at the TPS 44 A beamline of NSRRC, and by a home-made laboratory-based X-ray absorption spectrometer (Super XAFS). *In situ* Ni K- and Fe K-edge XAS (fluorescence mode) were measured at the BL14W1 beamline of the Shanghai Synchrotron Radiation Facility with a Si(111) double-crystal monochromator. Spectrum analysis was carried out using ATHENA and ARTEMIS (Demeter software package).

### *In Situ* Raman spectroscopy

Raman spectral data were collected by using a confocal microscope (Horiba, LabRAM HR Evolution). The source of the excitation was a 473 nm laser beam. The duration for acquiring spectra varied between 10 and 30 s. Calibration of spectra was carried out using a silicon wafer with a standard peak at 520.7 cm^–1^. A three-electrode setup was used in the *in situ* Raman spectroscopy measurements in 0.1 M KOH, including a working electrode of 5 mm glassy carbon, a counter electrode made of platinum wire, and a Hg/HgO reference electrode.

## Supporting Information Available

Details of DFT simulations, physicochemical characterization methods, optimization of the catalysts, TEM and SEM characterization before and after the OER, Raman spectroscopy, XAS simulations, XAS characterization, and ICP-MS analysis (PDF)
