# Supplementary material for: Balance between FeIV–NiIV synergy and Lattice Oxygen Contribution for Accelerating Water Oxidation
Source: ACS Nano. 2024 May 21;18(22):14496–506. doi: 10.1021/acsnano.4c01718 (PMC11155238; doi:10.1021/acsnano.4c01718)
Supplement: Supplementary file 1 — nn4c01718_si_001.pdf [file nn4c01718_si_001.pdf]

Balance between Fe^{IV}-Ni^{IV} synergy and Lattice Oxygen Contribution for Accelerating Water Oxidation

Chao Jing[†,○◆], *Lili Li*[‖◆], *Yi-Ying Chin*[⊥], *Chih-Wen Pao*[#], *Wei-Hsiang Huang*[#], *Miaomiao Liu*[†], *Jing Zhou*[∇], *Taotao Yuan*[‡], *Xiangqi Zhou*[†], *Yifeng Wang*[†,○], *Chien-Te Chen*[#], *Da-Wei Li*[‡*], *Jian-Qiang Wang*[†,○], *Zhiwei Hu*[§*], *Linjuan Zhang*[†,○*]

[†]Key Laboratory of Interfacial Physics and Technology, Shanghai Institute of Applied Physics, Chinese Academy of Sciences, Jialuo Road 2019, Shanghai 201800, P.R. China

[‡]School of Chemistry & Molecular Engineering, East China University of Science and Technology, 130 Meilong Road, Shanghai 200237, P.R. China

[§]Max Planck Institute for Chemical [‖]Physics of Solids, Nöthnitzer Strasse 40, Dresden 01187, Germany

[‖] State Key Laboratory of Crystal Materials and Institute of Crystal Materials, Shandong University, Jinan 250100, P.R. China

[⊥]Department of Physics, National Chung Cheng University, Chiayi 621301, Taiwan, R.O. China

[#]National Synchrotron Radiation Research Center, 101 Hsin-Ann Road, Hsinchu 300092, Taiwan, R.O. China

[∇]Zhejiang Institute of Photoelectronics & Zhejiang Institute for Advanced Light Source, Zhejiang Normal University, Jinhua, Zhejiang 321004, P.R. China

[○]University of Chinese Academy of Sciences, Beijing 100049, P.R. China

Preparation of Fe-free KOH. High purity $Ni(NO_3)_2\cdot 6H_2O$ (99.999%) was used for the purification of the KOH electrolyte.[1-3] A total of 2.0 g of nickel nitrate salt was dissolved in 5 mL of ultrapure water. Subsequently, 20 mL of 1 M KOH was added to induce the precipitation of nickel hydroxide. The resulting solution was subjected to agitation followed by centrifugation to separate the supernatant. The obtained nickel hydroxide was then subjected to washing using 2 mL of 1 M KOH and 20 mL of ultrapure water in three consecutive dispersing-centrifugation cycles. The resultant nickel hydroxide precipitate was suspended by adding 50 mL of 1 M KOH. After shaking for a minimum of 30 min, the mixture was allowed to settle undisturbed for at least 3 h. The subsequent centrifugation process yielded a purified KOH electrolyte, free from iron impurities.

Physicochemical characterization. Field emission scanning electron microscopy (SEM) images (Zeiss Merlin) were acquired at a voltage of 5 kV. Transmission electron microscopy (TEM) measurements were conducted using an FEI Tecnai G2 F20 S-TWIN electron microscope. High-resolution (HR) inductively coupled plasma mass spectrometry (ICP-MS) was analyzed on a Nu Attom instrument to detect the concentration of different elements.

Density functional theory (DFT) calculations. For this study, the Vienna ab initio simulation package (VASP)[4, 5] was used to perform the DFT calculations in conjunction with projector-augmented wave (PAW) formalism.[6] Consequently, The Ni $3d^8 4s^2$, Fe $3d^6 4s^2$, Al $3s^2 3p^1$, and O $2s^2 2p^4$ states were treated as valence electrons. The electronic

wave functions were expanded in plane waves using an energy cut-off of 500 eV, and the force and energy convergence criteria were set to 0.02 eV $Å^{-1}$ and 10^{-5} eV, respectively. The electron exchange and correlation within the generalized gradient approximation of the Perdew–Burke–Ernzerhof functional were used to optimize the configurations.[7] To accurately evaluate the electronic properties of NiO_2 (Ni), FeO_2 (Fe), Fe-doped NiO_2 ($Ni_{0.9}$-$Fe_{0.1}$), and Fe-Al-doped NiO_2 ($Ni_{0.75}$-$Fe_{0.1}$-$Al_{0.1}$ with 5% cation vacancy), the Hubbard U-model was employed to describe the strong correlation of the localized Ni 3d and Fe 3d states, and the values of U_{eff} were set to 5.5 and 4.0 eV, respectively, according to a previous study.[8, 9] The reciprocal space was sampled using a 5 × 4 × 4 Monkhorst–Pack k-point mesh for DFT + U calculations. The OER steps for AEM, LOV and MLOV routes are depicted as below:

AEM:

$$\text{M+OH}^- \rightarrow \text{M-OH+e}^- \quad (1)$$

$$\text{M-OH+OH}^- \rightarrow \text{M-O +H}_2\text{O+e}^- \quad (2)$$

$$\text{M-O+OH}^- \rightarrow \text{M-OOH+e}^- \quad (3)$$

$$\text{M-OOH+OH}^- \rightarrow \text{M+O}_2\text{+H}_2\text{O+e}^- \quad (4)$$

LOV:

$$\text{M-V}_\text{O}\text{+OH}^- \rightarrow \text{M-OH}_{\text{V}_\text{O}}\text{+e}^- \quad (5)$$

$$\text{M-OH}_{\text{V}_\text{O}}\text{+OH}^- \rightarrow \text{M-O}_{\text{V}_\text{O}}\text{+H}_2\text{O+e}^- \quad (6)$$

$$\text{M-O}_{\text{V}_\text{O}}\text{+OH}^- \rightarrow \text{M-OOH}_{\text{V}_\text{O}}\text{+e}^- \quad (7)$$

$$\text{M-OOH}_{\text{V}_\text{O}}\text{+OH}^- \rightarrow \text{M-V}_\text{O}\text{+O}_2\text{+H}_2\text{O+e}^- \quad (8)$$

MLOV:

$$\text{M-O}_{\text{V}_\text{O}}\text{+OH}^- \rightarrow \text{OH-M-O}_{\text{V}_\text{O}}\text{+e}^- \quad (9)$$

$$\text{HO-M-O}_{\text{V}_\text{O}}\text{+OH}^- \rightarrow \text{OO-M-V}_\text{O}\text{+H}_2\text{O+e}^- \quad (10)$$

$$\text{OO-M-V}_\text{O}\text{+OH}^- \rightarrow \text{M-OH}_{\text{V}_\text{O}}\text{+O}_2\text{+e}^- \quad (11)$$

$$\text{M-OH}_{\text{V}_\text{O}}\text{+OH}^- \rightarrow \text{M-O}_{\text{V}_\text{O}}\text{+H}_2\text{O+e}^- \quad (12)$$

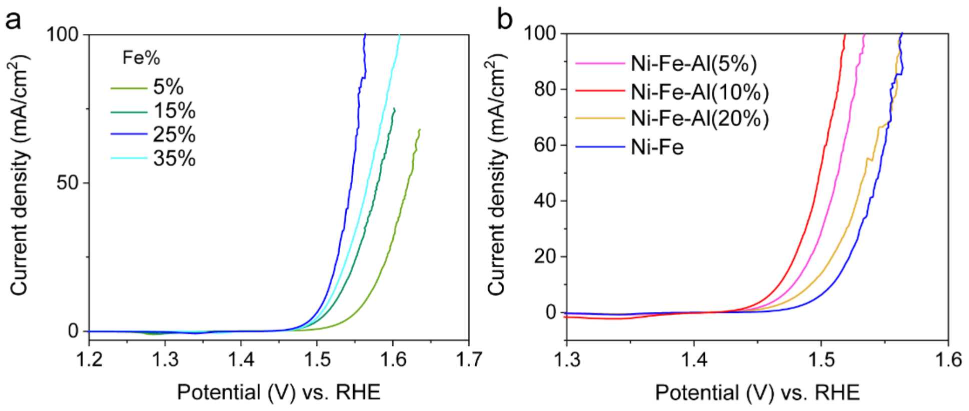

Figure S1. Ratio optimization of the doped Fe in Ni-Fe hydroxide (a) and doped Al in Ni-Fe-Al (b) hydroxide. Findings show that the doping of 25% Fe in Ni-Fe hydroxide and the doping of 10% Al in Ni-Fe (25%)-Al hydroxide yielded the best oxygen evolution reaction (OER) performance.

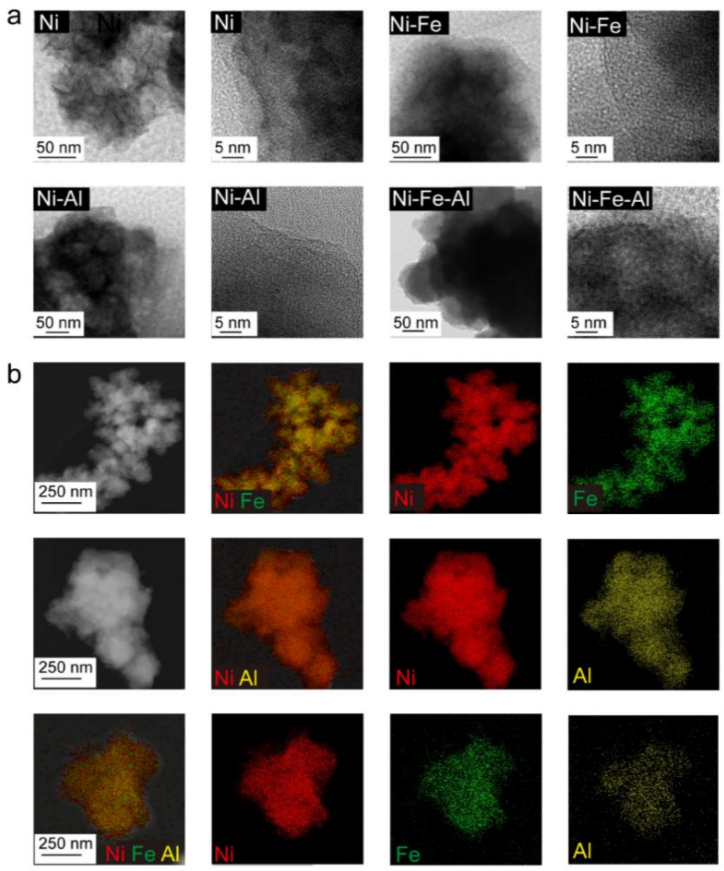

Figure S2. (a) Transmission electron microscopy (TEM) characterization of the Ni, Ni-Fe, Ni-Al, and Ni-Fe-Al hydroxides. (b) TEM mappings of the Ni-Fe, Ni-Al, and Ni-Fe-Al hydroxides.

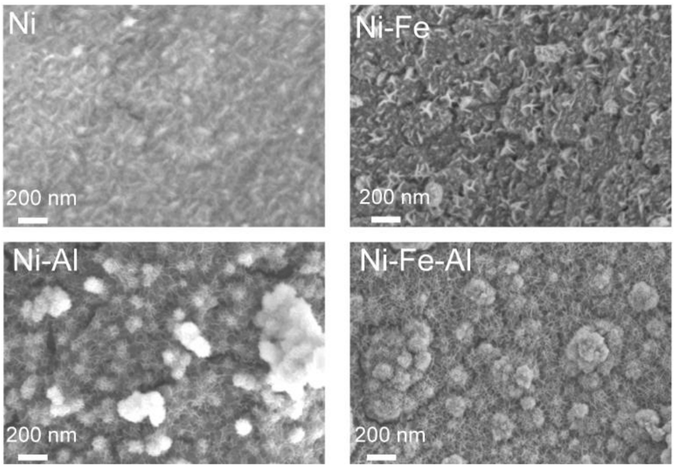

Figure S3. Scanning electron microscopy (SEM) characterization of the Ni, Ni-Fe, Ni-Al, and Ni-Fe-Al hydroxides.

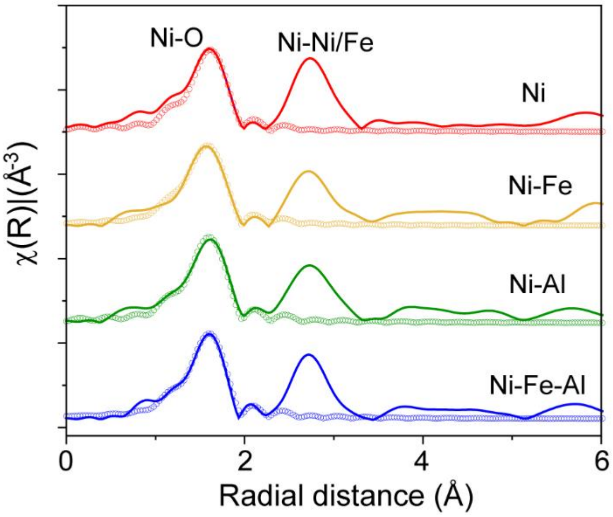

Figure S4. Fourier transformed $K^2\chi(R)$ Ni K-edge extended X-ray absorption fine structure (EXAFS) and corresponding simulation results of the pristine Ni, Ni-Fe, Ni-Al, and Ni-Fe-Al hydroxides.

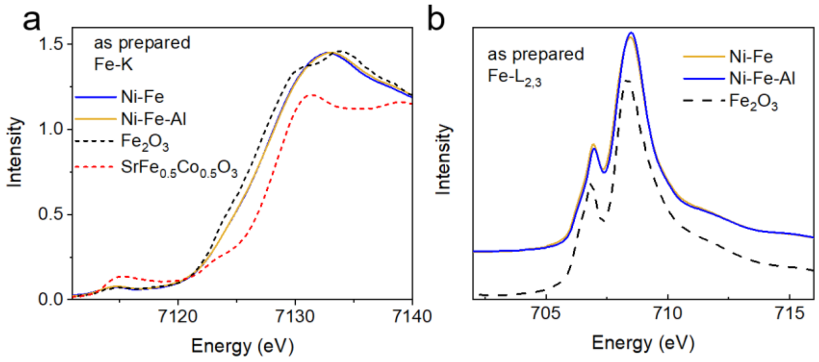

Figure S5. (a) Fe K-edge X-ray absorption spectroscopy (XAS) spectra of Ni-Fe and Ni-Fe-Al hydroxides with $Fe(III)_2O_3$ and $SrFe(IV)_{0.5}Co_{0.5}O_3$ references. (b) Fe L_3-edge XAS spectra of Ni-Fe and Ni-Fe-Al hydroxides with Fe_2O_3 reference.

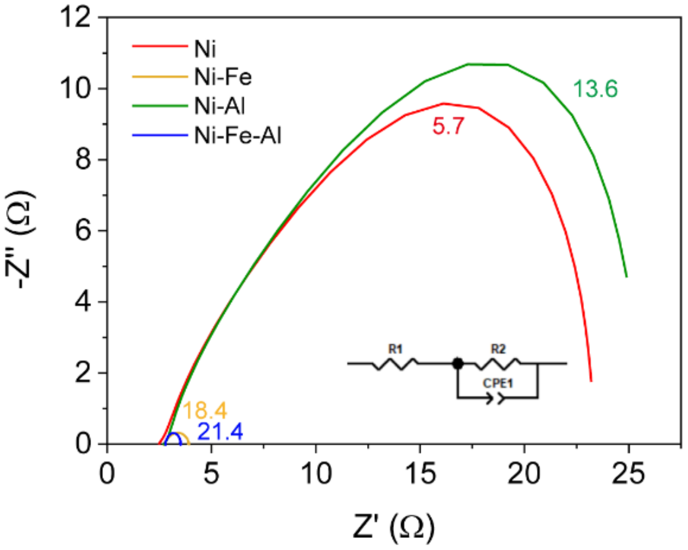

Figure S6. Nyquist plots of Ni, Ni-Fe, Ni-Al and Ni-Fe-Al hydroxides obtained at 1.56 V vs. RHE. Inset is the equivalent circuit. The calculated double-layer capacitance (C_{dl}) of Ni, Ni-Fe, Ni-Al and Ni-Fe-Al hydroxides are 5.7,18.4, 13.6, 21.4 mF cm^{-2}, respectively.

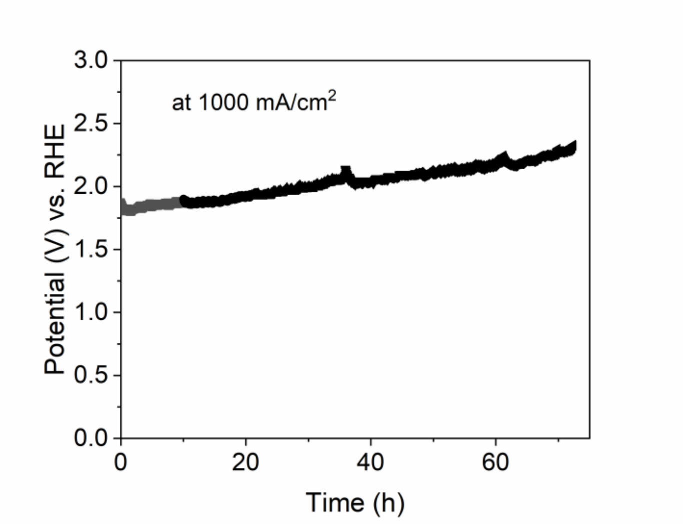

Figure S7. Stability test at 1000 mA cm^{-2} of Ni-Fe hydroxide using Ni foam as the working electrode (iR compensation: 90%).

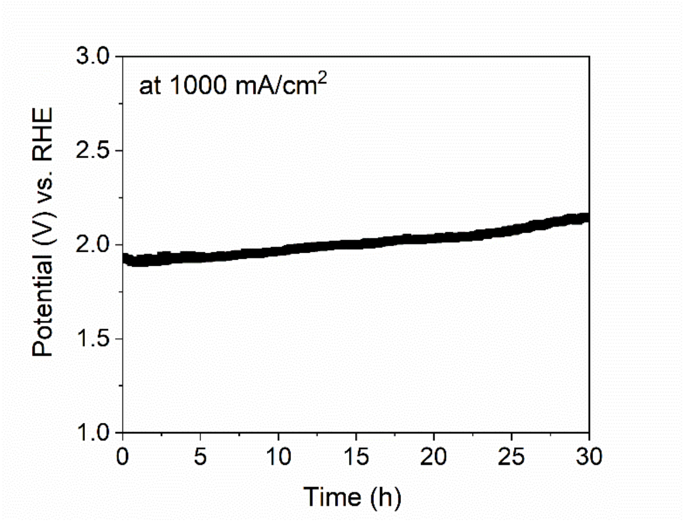

Figure S8. Stability test at 1000 mA cm^{-2} of bare Ni foam (iR compensation: 90%).

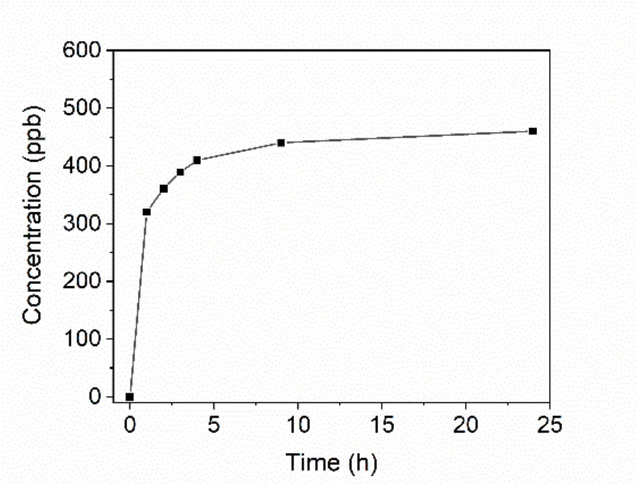

Figure S9. Concentration of dissolved Al ions of Ni-Fe-Al hydroxide during the OER process at 500 mA/cm^2 measured by ICP-MS.

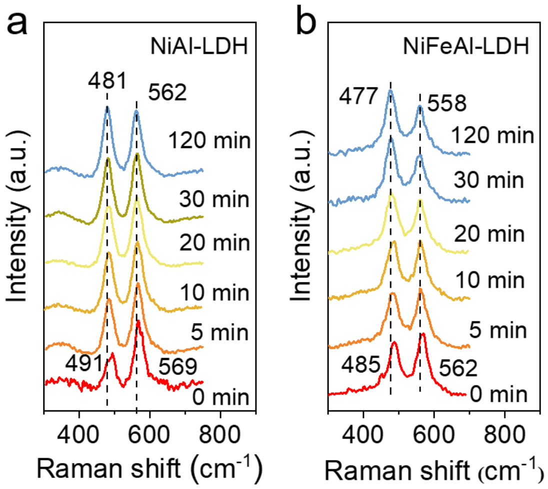

Figure S10. Raman spectra of Ni-Al (a), and Ni-Fe-Al (b) hydroxide catalysts under potential-off conditions after the OER in 0.1 M KOH electrolyte.

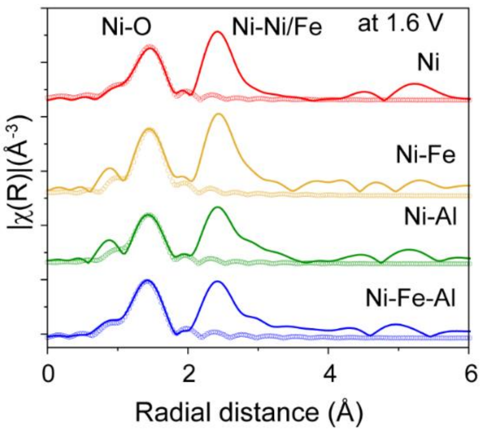

Figure S11. Fourier transformed $K^2\chi(R)$ Ni K-edge EXAFS and corresponding simulation results of the Ni, Ni-Fe, Ni-Al, and Ni-Fe-Al hydroxides during the OER process at 1.6 V vs. RHE in 0.1 M KOH electrolyte.

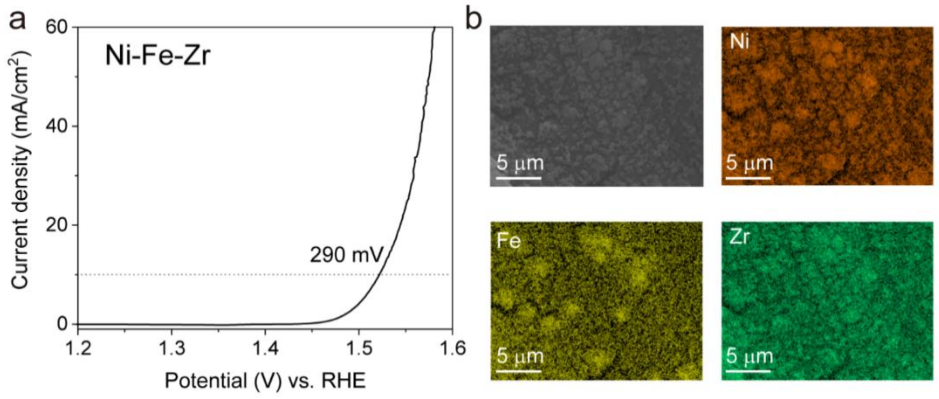

Figure S12. (a) Electrochemical performance of Zr doped Ni-Fe hydroxide in 1 M KOH electrolyte solution (scan rate: 5 mV s^{-1}, iR compensation: 90%). (b) SEM characterization of Zr doped Ni-Fe hydroxide with element mapping of Ni, Fe and Zr.

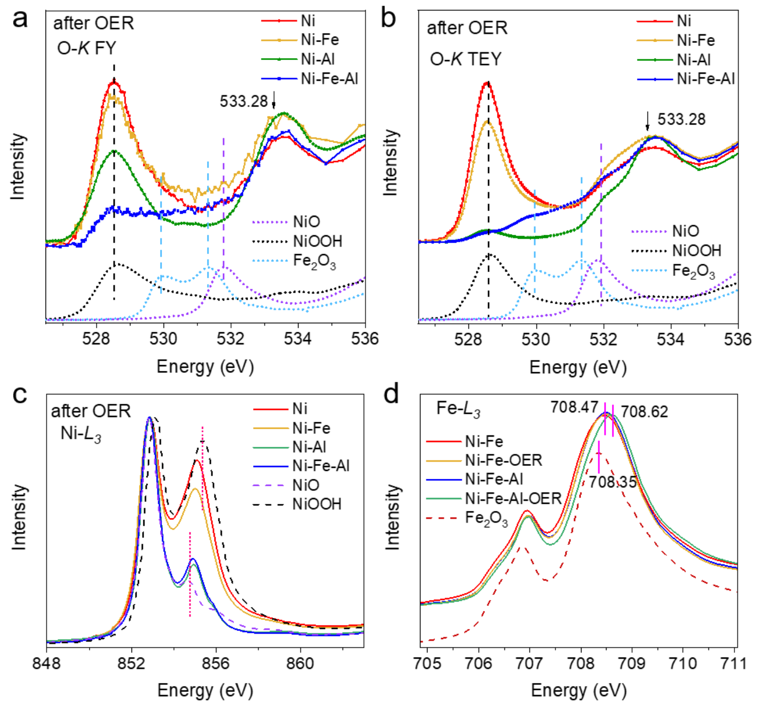

Figure S13. (a) Total electron yield (TEY) mode of the O-K edge and (b) Fluorescence yield (FY) mode of the O-K edge XAS spectra of Ni, Ni-Fe, Ni-Al, and Ni-Fe-Al hydroxide after the OER in 1 M KOH electrolyte (at 1.8 V vs. RHE for 2 h) with NiO, NiOOH, and Fe_2O_3 reference samples. (c-d) Ni L_3-edge and Fe L_3-edge XAS spectra of Ni, Ni-Fe, Ni-Al, and Ni-Fe-Al hydroxide after the OER in 1 M KOH electrolyte (at 1.8 V vs. RHE for 2 h) with NiO, NiOOH, and Fe_2O_3 reference samples.

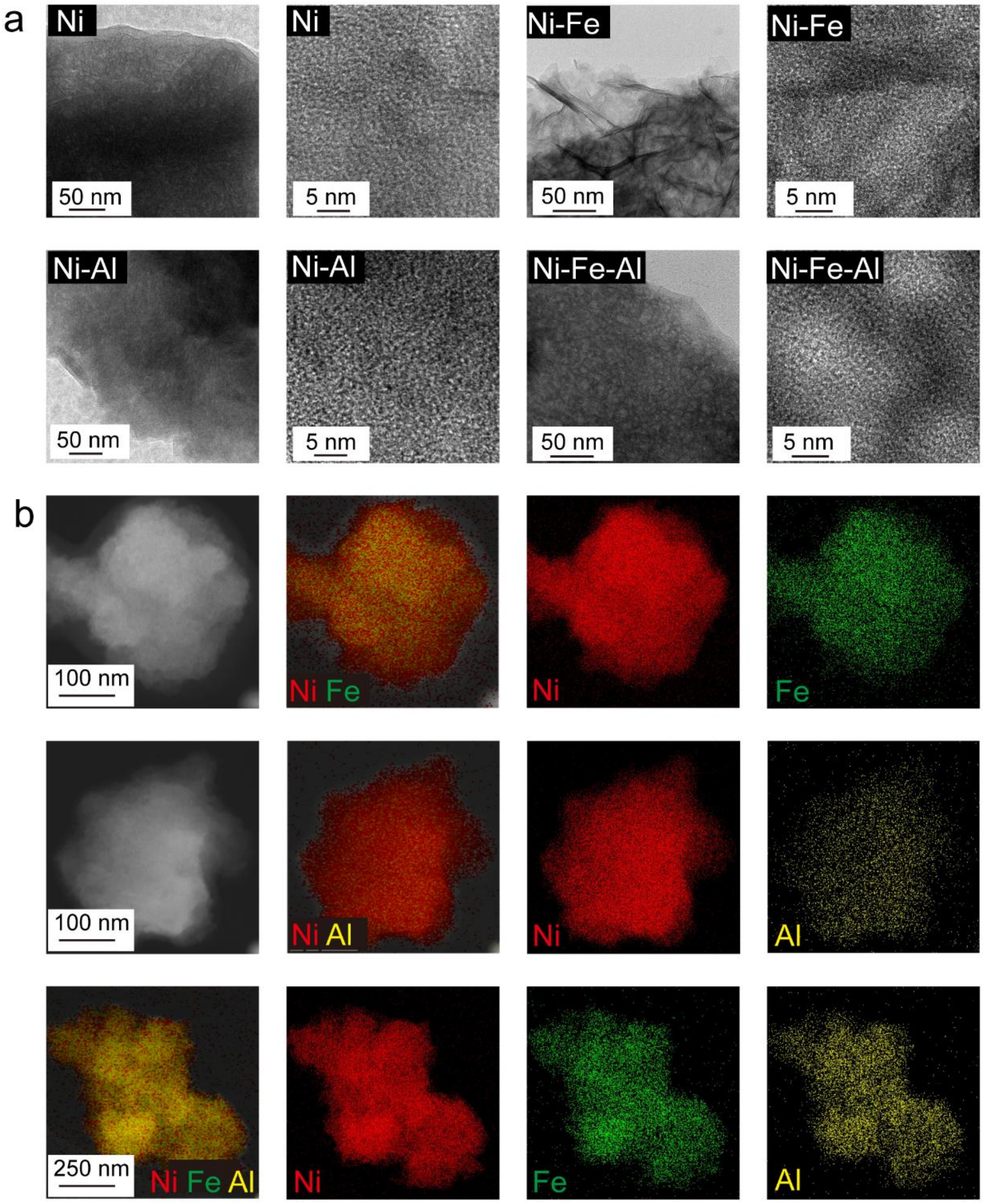

Figure S14. (a) TEM characterization of the Ni, Ni-Fe, Ni-Al, and Ni-Fe-Al hydroxides after the OER process at 1.8 V vs. RHE for 2 h in 1 M KOH electrolyte. (b) TEM mappings of Ni-Fe, Ni-Al, and Ni-Fe-Al hydroxides.

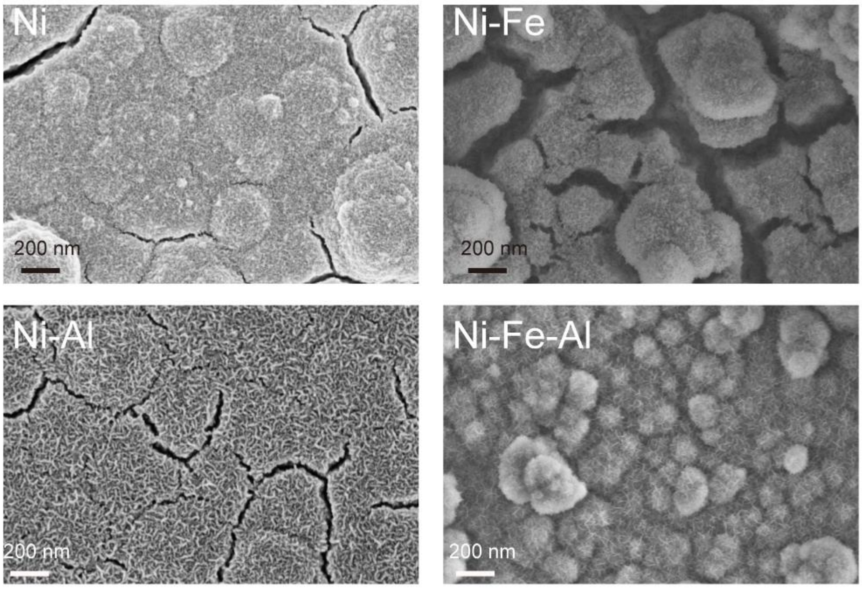

Figure S15. SEM characterization of the Ni, Ni-Fe, Ni-Al, and Ni-Fe-Al hydroxides after the OER process at 1.8 V vs. RHE for 2 h in 1 M KOH electrolyte.

Table S1. Ratios of different elements in the Ni-Fe, Ni-Al, and Ni-Fe-Al hydroxides before and after the OER process in 1 M KOH electrolyte (at 1.8 V vs. RHE for 2 h) measured by ICP-MS.

**Pristine catalysts**	**Ni%**	**Fe%**	**Al%**
Ni-Fe	89.97	10.03	
Ni-Al	87.96		12.04
Ni-Fe-Al	81.90	10.54	7.56
**After OER**	**Ni%**	**Fe%**	**Al%**
Ni-Fe	90.81	9.19	
Ni-Al	93.14		6.86
Ni-Fe-Al	83.69	11.58	4.73

Table S2. EXAFS simulation parameters for the as-prepared Ni, Ni-Fe, Ni-Al, and Ni-Fe-Al hydroxide catalysts.

**Samples**	**Shell**	**N**	**R (Å)**	**σ^2**	**R-factor**
**Ni**	Ni-O	5.7 ± 0.7	2.05	0.004	0.01
**Ni-Fe**	Ni-O	6.3 ± 0.4	2.03	0.006	0.004
**Ni-Al**	Ni-O	5.9 ± 0.3	2.04	0.004	0.002
**Ni-Fe-Al**	Ni-O	6.1 ± 0.4	2.04	0.005	0.004

Table S3. EXAFS simulation parameters for the Ni, Ni-Fe, Ni-Al, and Ni-Fe-Al hydroxide catalysts during the OER process at 1.6 V vs. RHE in 0.1 M KOH electrolyte.

Samples	Shell	N	R (Å)	σ^2	R-factor
**Ni**	Ni-O	4.4 ± 0.6	1.88	0.004	0.007
**Ni-Fe**	Ni-O	4.6 ± 0.4	1.88	0.004	0.006
**Ni-Al**	Ni-O	4.3 ± 0.3	1.88	0.004	0.005
**Ni-Fe-Al**	Ni-O	4.9 ± 0.4	1.87	0.005	0.003

References

1. Trotochaud, L.; Young, S. L.; Ranney, J. K.; Boettcher, S. W., Nickel-iron oxyhydroxide oxygen-evolution electrocatalysts: the role of intentional and incidental iron incorporation. *J. Am. Chem. Soc.* **2014,** *136* (18), 6744-53.
2. Burke, M. S.; Kast, M. G.; Trotochaud, L.; Smith, A. M.; Boettcher, S. W., Cobalt-iron (oxy)hydroxide oxygen evolution electrocatalysts: the role of structure and composition on activity, stability, and mechanism. *J. Am. Chem. Soc.* **2015,** *137* (10), 3638-48.
3. Moysiadou, A.; Lee, S.; Hsu, C. S.; Chen, H. M.; Hu, X., Mechanism of Oxygen Evolution Catalyzed by Cobalt Oxyhydroxide: Cobalt Superoxide Species as a Key Intermediate and Dioxygen Release as a Rate-Determining Step. *J. Am. Chem. Soc.* **2020,** *142* (27), 11901-11914.
4. Kresse, G.; Furthmüller, J., Efficient iterative schemes for ab initio total-energy calculations using a plane-wave basis set. *Phys. Rev. B* **1996,** *54* (16), 11169-11186.
5. Kresse, G.; Furthmüller, J., Efficiency of ab-initio total energy calculations for metals and semiconductors using a plane-wave basis set. *Comput. Mater. Sci.* **1996,** *6* (1), 15-50.
6. Kresse, G.; Joubert, D., From ultrasoft pseudopotentials to the projector augmented-wave method. *Phys. Rev. B* **1999,** *59* (3), 1758-1775.
7. Perdew, J. P.; Burke, K.; Ernzerhof, M., Generalized Gradient Approximation Made Simple. *Phys. Rev. Lett.* **1996,** *77* (18), 3865-3868.
8. Martirez, J. M. P.; Carter, E. A., Effects of the Aqueous Environment on the Stability and Chemistry of β-NiOOH Surfaces. *Chem. Mater.* **2018,** *30* (15), 5205-5219.
9. Gu, X.-K.; Nikolla, E., Design of Ruddlesden–Popper Oxides with Optimal Surface Oxygen Exchange Properties for Oxygen Reduction and Evolution. *ACS Catal.* **2017,** *7* (9), 5912-5920.
